# The Complete Plastid Genome of *Astrohibiscus caesius* (Hibisceae, Malvaceae) and Its Phylogenetic Placement

**DOI:** 10.1002/ece3.74010

**Published:** 2026-07-11

**Authors:** Rushan Yan, Furrukh Mehmood, Liya Guo, Mahpara Akhtar, Abdul Sammad, Jiahui Sun, Parviz Heidari, Abdullah, Xiaoxuan Tian

**Affiliations:** ^1^ State Key Laboratory of Chinese Medicine Modernization Tianjin University of Traditional Chinese Medicine Tianjin China; ^2^ Haihe Laboratory of Modern Chinese Medicine Tianjin China; ^3^ College of Biological Engineering Qingdao University of Science and Technology Qingdao China; ^4^ Department of Botany Women University of Azad Jammu and Kashmir Bagh Pakistan; ^5^ State Key Laboratory for Quality Ensurance and Sustainable Use of Dao‐di Herbs, National Resource Center for Chinese Materia Medica China Academy of Chinese Medical Sciences Beijing China; ^6^ Faculty of Agriculture Shahrood University of Technology Shahrood Iran

**Keywords:** *Astrohibiscus caesius*, Hibisceae, Malvaceae, phylogenomics, plastome, simple sequence repeats

## Abstract

Tribe Hibisceae (Malvaceae) has persistent taxonomic challenges due largely to the extensive polyphyly of *Hibiscus* L. Recently, *Hibiscus caesius* was segregated into the monotypic genus *Astrohibiscus* McLay and R.L. Barrett as *Astrohibiscus caesius* (Garcke) McLay and R.L. Barrett. Here, we sequenced, *de novo* assembled, and characterized the first complete plastid genome of *A. caesius* and conducted phylogenomic analyses to provide a genomic resource for evolutionary, phylogeographic, and conservation studies of this newly recognized genus. The plastid genome is 161,092 bp in length and exhibits the typical quadripartite architecture, comprising a large single‐copy region (89,340 bp), a small single‐copy region (20,486 bp), and a pair of inverted repeats (25,633 bp each). The genome has an overall GC content of 36.94% and encodes 112 unique genes (78 protein‐coding, 30 tRNA, and 4 rRNA genes). Codon usage showed bias toward A/T‐ending codons (RSCU > 1), while leucine was the most abundant amino acid, and cysteine was the least frequent. Simple sequence repeat (SSR) analysis identified a predominance of A/T‐rich mononucleotide repeats, providing candidate molecular markers for population‐genetic studies. Maximum likelihood phylogenetic analysis of 78 shared protein‐coding genes across subfamily Malvoideae yielded a well‐resolved topology with strong bootstrap support. *Hibiscus* was polyphyletic, consistent with previous studies, while *A. caesius* formed a distinct lineage outside all major *Hibiscus* clades, consistent with its recognition as a distinct genus. These findings provide a genomic resource for future evolutionary, phylogeographic, and taxonomic studies within Hibisceae.

## Graphical abstract

The complete plastid genome of *Astrohibiscus caesius* has a typical quadripartite structure (161,092 bp) and encodes 112 unique genes with an AT‐rich bias. Phylogenomic analysis confirms that *Hibiscus* is polyphyletic and *Astrohibiscus* forms a distinct lineage outside all major *Hibiscus* clades, providing whole‐plastome evidence supporting this monotypic genus.

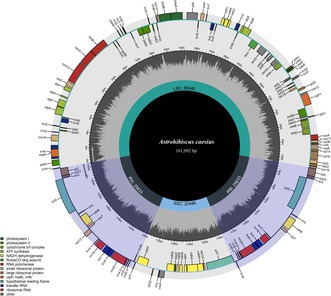

## 1 Introduction

The plastid genome (plastome) is a circular, double‐stranded DNA molecule typically 120–170 kb in length, organized into a conserved quadripartite structure comprising a large single‐copy (LSC) region, a small single‐copy (SSC) region, and a pair of inverted repeats (IRa and IRb) (Palmer 1985; Daniell et al. 2016). In most angiosperms, the plastome encodes approximately 112 unique genes, including protein‐coding genes, transfer RNAs (tRNAs), and ribosomal RNAs (rRNAs). The plastome is widely used for phylogenetics, barcoding, population genetics, and conservation genetics (Daniell et al. 2016; Abdullah, Li, et al. 2026; Zeng et al. 2025).

Tribe Hibisceae (Malvoideae, Malvaceae) comprises approximately 43 genera distributed across tropical and subtropical regions worldwide, following a comprehensive revision that substantially restricted *Hibiscus* to the/Euhibiscus lineage (Hanes et al. 2024). The tribe taxonomy has long been complicated by the polyphyly of the broadly defined *Hibiscus* L., with allied genera such as *Abelmoschus* Medik., *Malvaviscus* Fabr., *Kosteletzkya* C. Presl, *Urena* Dill. ex L., *Kydia* Roxb., and *Pavonia* Cav. (Pfeil and Crisp 2005; Hanes et al. 2024; Abdullah, Chen, et al. 2026; Shi et al. 2026). Hanes et al. (2024) established the monotypic *Astrohibiscus* McLay & R.L. Barrett for *Hibiscus caesius* Garcke and named *Astrohibiscus caesius* (Garcke) McLay and R.L. Barrett. The species is a perennial shrub distributed from southwestern Tanzania to South Africa and from Pakistan to India, occurring in seasonally dry tropical woodlands, riverine margins, and disturbed areas (POWO 2026).

Plastome resources have been reported for many *Hibiscus* species and other Malvaceae (Abdullah et al. 2019, 2020, 2021; Abdullah, Chen, et al. 2026; Alzahrani 2021; Lei et al. 2025; Shi et al. 2026; Wang et al. 2021; Yan et al. 2025, 2026; Zhang et al. 2025). However, no plastid genome has been reported for the newly established genus *Astrohibiscus*. Here, we report the *de novo* assembled complete plastome of *A. caesius* to provide genomic resources for this newly recognized genus, characterize its plastome structure and gene content, and reconstruct its phylogenetic placement within Hibisceae.

## 2 Materials and Methods

### 2.1 Sample Collection, DNA Extraction and Sequencing

Leaf material of *Astrohibiscus caesius* was collected from the herbarium of the Pakistan Museum of Natural History, Islamabad, Pakistan, under voucher PMNH2151. Total genomic DNA was extracted from 15 mg of dried leaf tissue using the Plant Genomic DNA Kit (TIANGEN BIOTECH, Beijing, China) following the manufacturer's protocol. DNA quality and concentration were evaluated using an Agilent 5400 System (Agilent Technologies, Santa Clara, CA, USA). A sequencing library with a 350‐bp insert size was prepared, and paired‐end sequencing (2 × 150 bp) was performed on an Illumina NovaSeq 6000 platform at Novogene (Tianjin, China).

### 2.2 Plastid Genome Assembly and Annotation

Plastid genome assembly and quality assessment were conducted using CGAS v1.0.1 Module 1 (Abdullah, Yan, and Tian 2026), which integrates read quality control, genome assembly, and coverage depth analysis. Raw reads were filtered using fastp v1.0.1 (Chen 2025) as implemented in CGAS. Filtered reads were assembled *de novo* using GetOrganelle v1.7.7.1 (Jin et al. 2020) with multiple k‐mer sizes (21, 45, 65, 85, and 105) as configured in CGAS. The genome was assembled as two circular contigs that differed only in the orientation of the SSC region, confirming a complete circular assembly. The genome assembly and IR boundary positions were further validated by remapping the raw reads to the assembled genome using BWA‐MEM (Li and Durbin 2009), and coverage depth was calculated with SAMtools (Danecek et al. 2021), both integrated within CGAS Module 1. The assembled genome was annotated using GeSeq v2.0.3 (Tillich et al. 2017), and tRNA genes were predicted and verified using both ARAGORN v1.2.38 (Laslett and Canback 2004) and tRNAscan‐SE v2.0.7 (Chan and Lowe 2019). Annotations were further refined and validated using PGA (Plastid Genome Annotator) (Qu et al. 2019), implemented through CGAS Module 2, with *Hibiscus rosa‐sinensis* (NC042239) as the reference genome. The annotations were additionally verified by pairwise alignment with the *H. rosa‐sinensis* reference genome in Geneious Prime 2025, followed by manual inspection. A genome map was generated with Chloroplot (Zheng et al. 2020). The submission files to the National Center for Biotechnology Information (NCBI) were prepared using CGAS (Abdullah, Yan, and Tian 2026).

### 2.3 Analysis of Codon Usage, Amino Acid Frequency, and Microsatellites

Relative synonymous codon usage (RSCU), amino acid frequency, and simple sequence repeats (SSRs) were analyzed using CGAS (Abdullah, Yan, and Tian 2026). SSRs were identified using the following minimum repeat thresholds: 10 for mononucleotides, 5 for dinucleotides, 4 for trinucleotides, and 3 for tetra‐, penta‐, and hexanucleotides.

### 2.4 Phylogenetic Analysis

Complete plastome sequences representing distinct genera of Malvoideae were retrieved from the NCBI GenBank database. Protein‐coding genes were extracted using CGAS Module 14 (Abdullah, Yan, and Tian 2026), and each CDS was aligned individually with MAFFT v7.525 (Nakamura et al. 2018). The gene‐wise alignments were then concatenated into a single supermatrix of 78 shared protein‐coding genes, and partition files were generated for downstream analysis. Maximum‐likelihood (ML) phylogenetic analysis was performed using IQ‐TREE v3.0.1 (Wong et al. 2026), with *Bombax ceiba* (NC037494) designated as the outgroup. The best‐fit substitution model, TVM + F + I, was selected using ModelFinder (implemented in IQ‐TREE) under the Bayesian Information Criterion (BIC). Branch support was evaluated with 10,000 ultrafast bootstrap (UFBoot) replicates and 10,000 SH‐aLRT replicates. The resulting phylogenetic tree was visualized using the Interactive Tree of Life (iTOL v7) (Letunic and Bork 2024).

## 3 Results and Discussion

### 3.1 Characterization of the Plastid Genome of *Astrohibiscus caesius*

**FIGURE 1:**
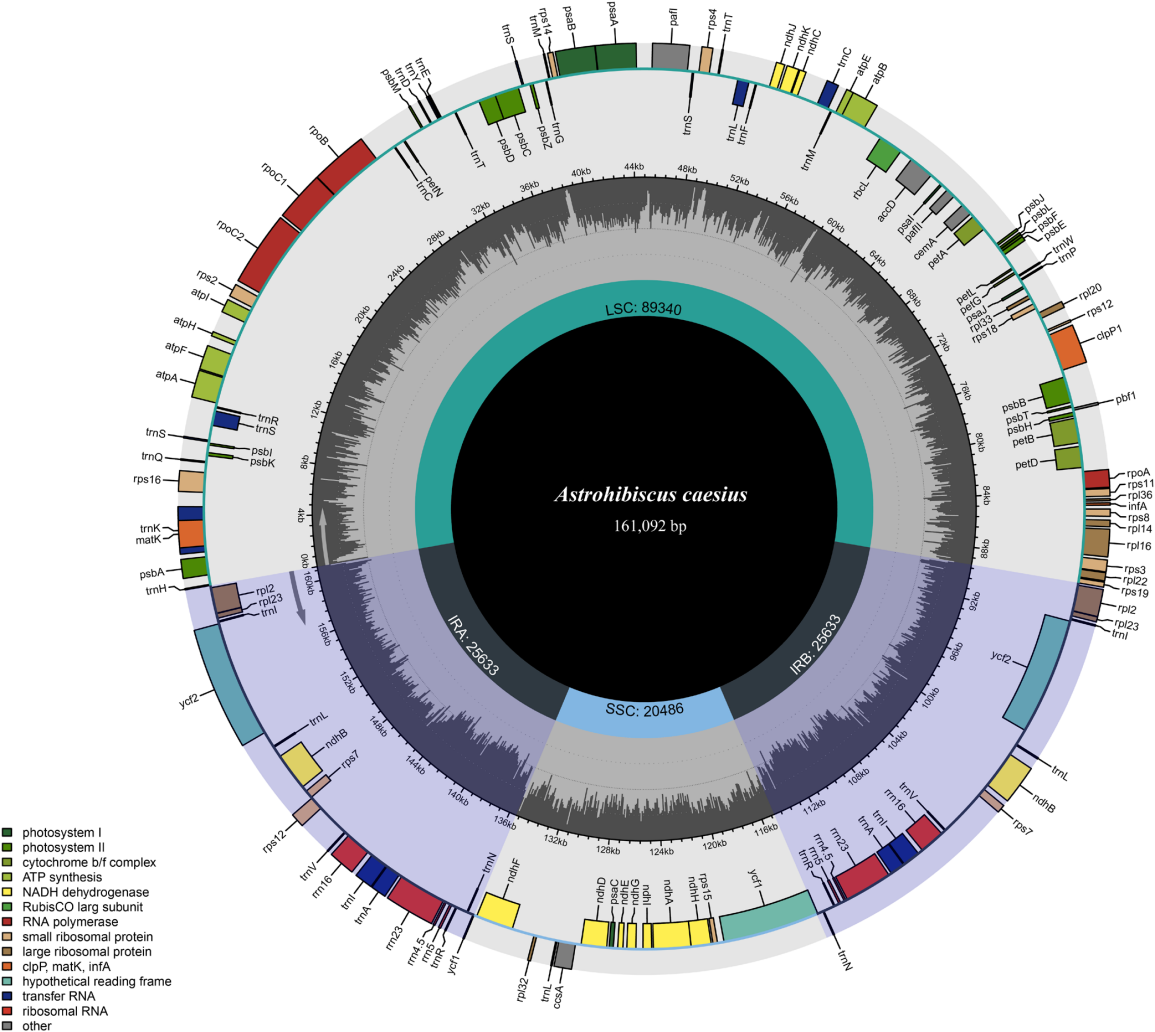
Circular map of the plastome of *Astrohibiscus caesius*. Genes inside the circle are transcribed clockwise, whereas those outside are transcribed counterclockwise. Color coding denotes distinct functional gene categories. The inner gray histogram depicts GC content variation across the genome.

Illumina sequencing of *A. caesius* yielded approximately 5.44 Gb of raw data, yielding 13.62 million paired‐end clean reads (2 × 150 bp). *De novo* assembly of 0.47 million reads, representing 3.5% of the total clean reads, produced a complete, circular plastome of 161,092 bp with a mean sequencing depth of 457×, confirming high assembly reliability (Figure S1). The IR boundaries displayed well‐connected junctions, confirming the integrity of the assembled plastome. The plastome exhibited the typical quadripartite architecture consisting of a LSC region (89,340 bp), a SSC region (20,486 bp), and two IR regions (IRa/IRb; 25,633 bp each) (Figure 1). Previously reported genome lengths for *Hibiscus* species vary between 159,125 and 163,360 bp (Abdullah, Chen, et al. 2026); the genome length of *A. caesius* falls within this range, and its conserved quadripartite organization is characteristic of most land plant plastomes and is consistent with reports from *Hibiscus*, Hibisceae, and other Malvaceae (Zhang et al. 2025; Yan et al. 2025; Kwon et al. 2023; Abdullah et al. 2020).

The overall GC content was 36.94%, with regional variation across the three partitions: LSC (34.84%), IRs (42.85%), and SSC (31.26%). Among functional gene classes, rRNA genes exhibited the highest GC content (55.47%), followed by tRNA genes (52.84%) and protein‐coding genes (38.13%). The elevated GC content of the IR regions is attributable primarily to the presence of GC‐rich rRNA genes and likely reflects purifying selection maintaining structural and functional conservation in these duplicated segments. The genome size and GC composition are consistent with values reported for other Hibisceae (Abdullah et al. 2020; Abdullah, Chen, et al. 2026; Shi et al. 2026).

The plastome encodes 112 unique genes: 78 protein‐coding genes, 30 tRNA genes, and four rRNA genes (Table 1). Sixteen genes were duplicated within the IR regions, including five protein‐coding genes (*ndhB*, *rpl2*, *rpl23*, *rps7*, and *ycf2*), four rRNA genes (*rrn16S*, *rrn23S*, *rrn4.5S*, and *rrn5S*), and seven tRNA genes (*trnA‐UGC*, *trnI‐GAU*, *trnL‐CAA*, *trnN‐GUU*, *trnR‐ACG*, *trnV‐GAC*, and *trnI‐CAU*). IR‐mediated gene duplication is a conserved feature that contributes to genome stability through functional redundancy. Eighteen genes contained introns: 12 protein‐coding genes (*atpF*, *petB*, *rps16*, *rps12*, *rpl2*, *ndhA*, *rpl16*, *ndhB*, *rpoC1*, *ycf3*, *petD*, and *clpP*) and six tRNA genes (*trnV‐UAC*, *trnK‐UUU*, *trnG‐UCC*, *trnI‐GAU*, *trnL‐UAA*, and *trnA‐UGC*), with *ycf3* and *clpP* each harboring two introns. Notably, *rps12* is trans‐spliced, with exon 1 in the LSC and exons 2–3 in the IR. Overall, the gene content and structural organization of the *A. caesius* plastome conform to the conserved pattern reported for Hibisceae and Malvaceae more broadly (Abdullah, Chen, et al. 2026; Shi et al. 2026; Yan et al. 2026; Zhang et al. 2025; Zhong et al. 2024), reflecting deep evolutionary conservation of plastome architecture.

**TABLE 1:** Gene content of the plastome of *Astrohibiscus caesius*.

Category for genes	Group of genes	Name of genes	Total
Self‐replication	Large subunit of ribosome	*rpl14*, rpl16*, rpl2* ^*a*^, *rpl20*, *rpl22*, *rpl23* ^*a*^, *rpl32*, *rpl33*, *rpl36*	11
	Small subunit of ribosome	*rps11*, rps12*, *rps14*, *rps15*, rps16*, *rps18*, *rps19*, *rps2*, *rps3*, *rps4*, *rps7* ^*a*^, *rps8*	13
	DNA dependent RNA polymerase	*rpoA*, *rpoB*, rpoC1*, *rpoC2*	4
	rRNA genes	*rrn16* ^*a*^, *rrn23* ^*a*^, *rrn4.5* ^*a*^, *rrn5* ^*a*^	8
	tRNA genes	trnA‐UGC* ^,*a*^, trnC‐ACA*, *trnC‐GCA*, *trnD‐GUC*, *trnE‐UUC*, *trnF‐GAA*, *trnG‐GCC*, *trnH‐GUG*, *trnI‐CAU* ^*a*^, trnI‐GAU*, trnK‐UUU*, *trnL‐CAA* ^*a*^, trnL‐UAA*, *trnL‐UAG*, *trnfM‐CAU, trnM‐CAU*, *trnN‐GUU* ^*a*^, *trnP‐UGG*, *trnQ‐UUG*, *trnR‐ACG* ^*a*^, *trnR‐UCU*, trnS‐CGA*, *trnS‐GCU*, *trnS‐GGA*, *trnS‐UGA*, *trnT‐GGU*, *trnT‐UGU*, *trnV‐GAC* ^*a*^, *trnW‐CCA*, *trnY‐GUA*	37
Photosynthesis	Photosystem I	*psaA*, *psaB*, *psaC*, *psaI*, *psaJ*	5
	Photosystem II	*psbA*, *psbB*, *psbC*, *psbD*, *psbE*, *psbF*, *psbH*, *psbI*, *psbJ*, *psbK*, *psbL*, *psbM*, *psbN (pbf1)*, *psbT*, *psbZ (lhbA)*	15
	NADPH dehydrogenase	ndhA*, ndhB* ^,*a*^, *ndhC*, *ndhD*, *ndhE*, *ndhF*, *ndhG*, *ndhH*, *ndhI*, *ndhJ*, *ndhK*	12
	Cytochrome b/f complex	*petA*, petB*, petD*, *petG*, *petL*, *petN*	6
	Subunits of ATP synthase	*atpA*, *atpB*, *atpE*, atpF*, *atpH*, *atpI*	6
	Large subunit of Rubisco	*rbcL*	1
	Photosynthesis assembly genes	ycf3 (pafI)*, *ycf4 (pafII)*	2
Other genes	Protease	clpP*	1
	Maturase	*matK*	1
	Envelope membrane protein	*cemA*	1
	Subunit of Acetyl‐CoA‐carboxylase	*accD*	1
	C‐type cytochrome synthesis gene	*ccsA*	1
	Conserved open reading frames	*ycf1, ycf2* ^*a*^	3
	Pseudogenes	*infAΨ*	1
		**Total number of genes**	129

*Note:* *, ^a^, and Ψ indicate genes containing introns, duplicated genes in inverted repeat (IR) regions, and pseudogenes, respectively. The *rps12* gene is a trans‐spliced gene and is not marked as duplicated despite appearing in multiple locations.

### 3.2 Amino Acid Frequency, Codon Usage and Microsatellite Analysis

**FIGURE 2:**
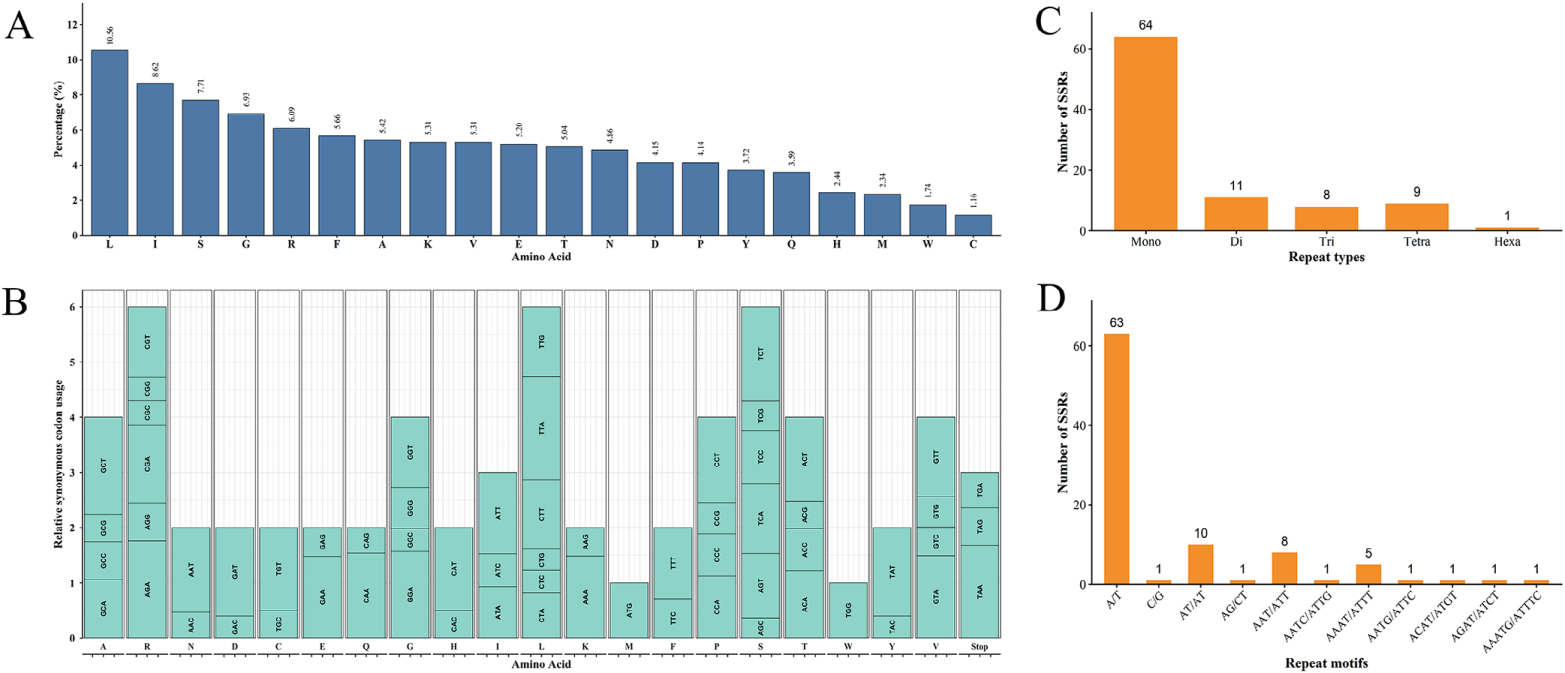
Analysis of amino acid frequency, codon usage, and simple sequence repeats (SSRs) in the plastome of *Astrohibiscus caesius*. (A) Relative percentage of each amino acid in the encoded proteome. (B) Relative synonymous codon usage (RSCU) values for all amino acids. (C) Number of SSRs classified by repeat type. (D) Frequency of different SSR motifs.

Amino acid frequency analysis revealed leucine as the most abundant residue, while cysteine was the least represented (Figure 2A). Relative synonymous codon usage (RSCU) analysis identified 30 codons with RSCU values > 1. Of these, 13 terminated in A, 16 in T, and only 1 in G, whereas no codon ending in C exhibited RSCU > 1. This pattern reveals a pronounced bias toward A/T‐ending codons at the synonymous third codon position, coupled with a marked underrepresentation of C/G‐ending codons (RSCU < 1) (Figure 2B). This A/T‐ending bias mirrors the genome‐wide AT‐rich composition and may have co‐evolved with tRNA availability and translational efficiency in the plastome. Analogous codon usage patterns have been documented in *Hibiscus*, tribe Hibisceae, and across other Malvaceae species (Abdullah et al. 2026; Yan et al. 2026; Shi et al. 2026; Zhong et al. 2024).

SSR analysis identified 93 repeat loci (Table S1), with mononucleotide repeats constituting the dominant class (64), followed by dinucleotide (11) and tetranucleotide (9) repeats; no pentanucleotide repeats were detected (Figure 2C). The majority of SSR motifs were A/T‐rich (Figure 2D), consistent with the AT‐biased nucleotide composition of the plastome. These findings are in accord with SSR distribution patterns reported for other Malvaceae plastomes (Abdullah, Li, et al. 2026; Shi et al. 2026; Geng et al. 2024; Zhong et al. 2024) and suggest that these loci may serve as informative markers for population‐genetic and phylogeographic studies of *A. caesius* .

### 3.3 Phylogenetic Analysis

**FIGURE 3:**
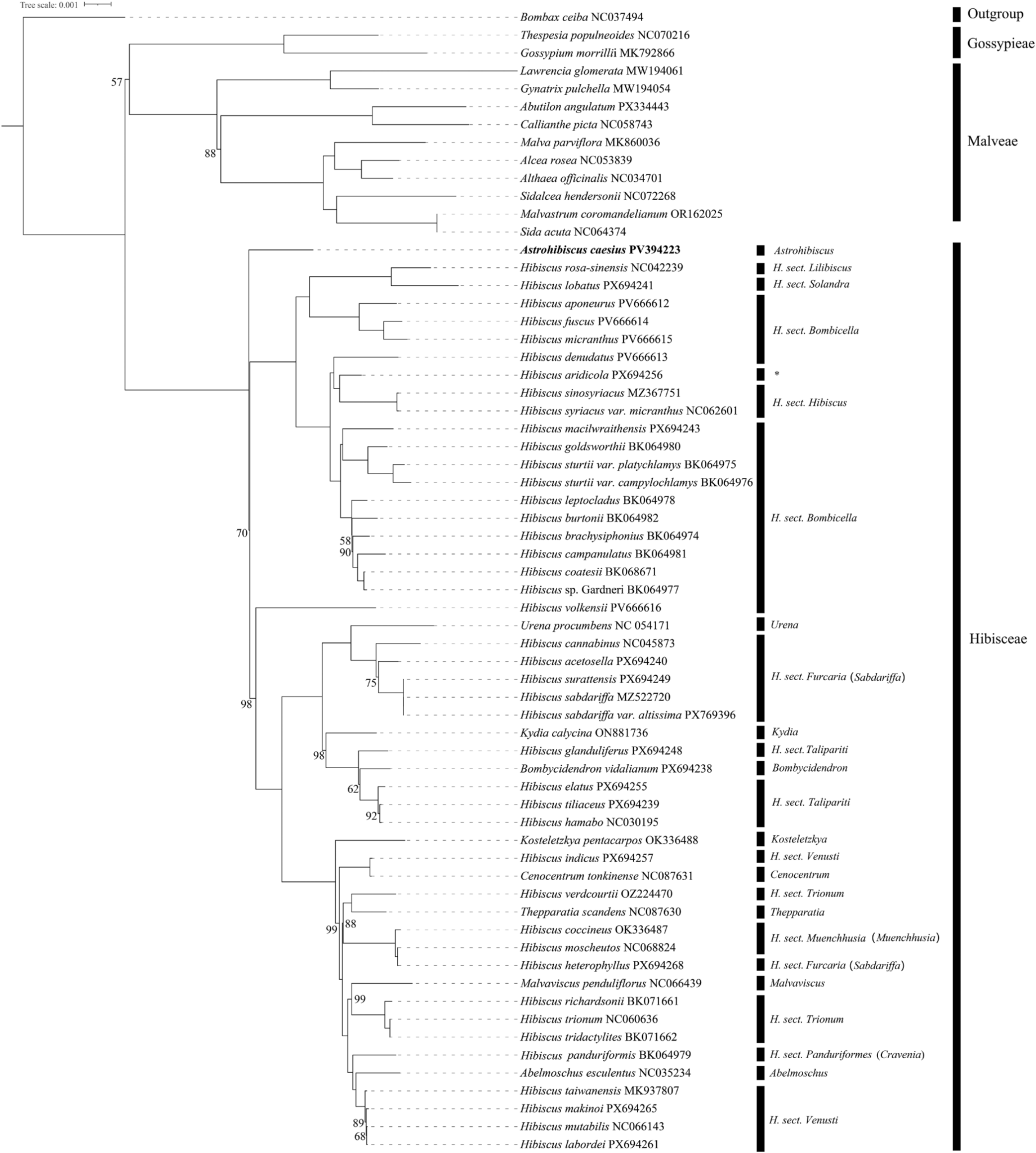
Maximum likelihood phylogenetic tree of tribe Hibisceae based on 78 shared protein‐coding genes. Ultrafast bootstrap support values below 100% are shown at the corresponding nodes. The plastome of *Astrohibiscus caesius* generated in this study is highlighted in bold.

The ML phylogenetic analysis recovered a well‐resolved topology with strong statistical support at key nodes (Figure 3). The majority of nodes received ultrafast bootstrap (UFBoot) support of 100%, which is consistent with previous plastome‐level reports (Abdullah, Chen, et al. 2026; Shi et al. 2026). This result demonstrates that the concatenated protein‐coding gene dataset provides robust phylogenetic signal for resolving evolutionary relationships within the tribe. Tribe Hibisceae was recovered as a strongly supported monophyletic group (UFBoot = 100%). However, *Hibiscus* was recovered as polyphyletic, with its species distributed across multiple non‐sister lineages. This polyphyletic pattern is congruent with earlier molecular studies (Small 2004; Pfeil and Crisp 2005; Hanes et al. 2024) and recent plastome‐based phylogenomic analyses (Abdullah, Chen, et al. 2026; Shi et al. 2026).

*Astrohibiscus caesius* (formerly *Hibiscus caesius*) was recovered as a distinct, well‐supported lineage (UFBoot = 100%) outside all major *Hibiscus* clades in the whole‐plastome phylogeny. This result is consistent with the establishment of *Astrohibiscus* by Hanes et al. (2024). Beyond its taxonomic implications, the plastome reported here provides a genomic resource for phylogeographic and biogeographic investigations of this species across its broad Afro‐Asian distribution.

The phylogenetic complexity of *Hibiscus* and its allied genera has been attributed to multiple evolutionary processes. Previous studies have suggested that incomplete lineage sorting associated with rapid diversification, together with hybridization and introgression, have contributed to phylogenetic discordance within the tribe (Shi et al. 2026). These evolutionary processes have complicated the delimitation of genera and contributed to the long‐recognized polyphyly of *Hibiscus* (Pfeil and Crisp 2005; Hanes et al. 2024). Our plastome phylogeny is consistent with these previous studies by recovering the complex evolutionary structure of the tribe and supporting the distinct placement of *Astrohibiscus*. Nevertheless, because plastomes are typically maternally inherited in Malvaceae and represent a single, non‐recombining genomic compartment, they capture only part of the evolutionary history. Consequently, incongruence with nuclear phylogenies resulting from incomplete lineage sorting, hybridization, or other reticulate evolutionary processes cannot be excluded. Future studies integrating nuclear single‐copy loci with plastome data will therefore be important for further refining generic relationships within Hibisceae and Malvaceae.

## 4 Conclusion

We report the first complete plastome of *Astrohibiscus caesius* (161,092 bp), which displays the conserved quadripartite structure and gene content typical of Malvaceae. Phylogenomic analysis placed *A. caesius* outside the core *Hibiscus* clades (UFBoot = 100%), consistent with its segregation as a distinct genus by Hanes et al. (2024). Our results also reflect the well‐documented polyphyly of *Hibiscus* sensu lato. The plastome and SSR markers presented here provide a foundation for future phylogeographic, population‐genetic, and comparative studies within Hibisceae.

## Author Contributions

**Rushan Yan:** conceptualization (equal), data curation (equal), formal analysis (equal), writing – original draft (equal). **Furrukh Mehmood:** conceptualization (equal), data curation (equal), formal analysis (equal), writing – original draft (equal). **Liya Guo:** data curation (equal), formal analysis (equal). **Mahpara Akhtar:** data curation (equal), formal analysis (equal). **Abdul Sammad:** data curation (equal), formal analysis (equal). **Jiahui Sun:** investigation (equal), methodology (equal). **Parviz Heidari:** resources (equal), supervision (equal), writing – review and editing (equal). **Abdullah:** conceptualization (equal), data curation (equal), formal analysis (equal), resources (equal), writing – review and editing (equal). **Xiaoxuan Tian:** investigation (equal), methodology (equal), resources (equal), supervision (equal), writing – review and editing (equal).

## Funding

This study was supported by Key project at central government level: The ability establishment of sustainable use for valuable Chinese medicine resources (2060302).

## Conflicts of Interest

The authors declare no conflicts of interest.

## Supporting information


**Figure S1:** Depth distribution of the *Astrohibiscus caesius* plastome. (A) Sequencing depth across the genome position. (B) Frequency of sequencing depth. The red dashed line indicates the mean depth of 457×.

**Table S1:** Distribution and comparative characteristics of SSRs in the plastome of *Astrohibiscus caesius*.

## Data Availability

The assembled plastid genome sequence has been deposited in NCBI under accession number PV394223; the associated raw sequencing data are available under BioProject PRJNA1332951.
