# Supplementary figures and images for: The Complete Plastid Genome of Astrohibiscus caesius (Hibisceae, Malvaceae) and Its Phylogenetic Placement
Source: Ecol Evol. 2026 Jul 11;16(7):e74010. doi: 10.1002/ece3.74010 (PMC13355160; doi:10.1002/ece3.74010)

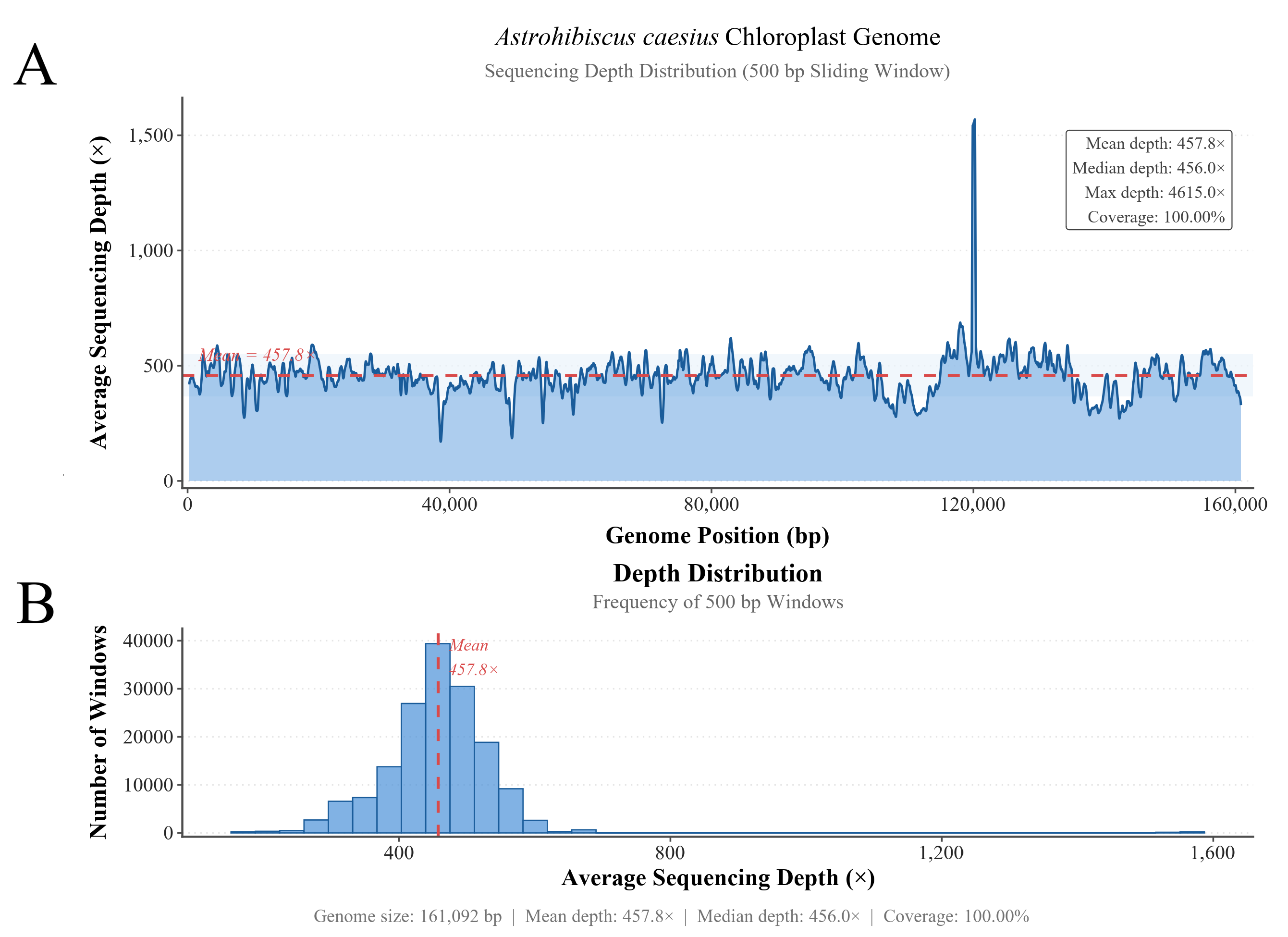

Supplement: Supplementary file 1 — Figure S1: Depth distribution of the Astrohibiscus caesius plastome. (A) Sequencing depth across the genome position. (B) Frequency of sequencing depth. The red dashed line indicates the mean depth of 457×. [file ECE3-16-e74010-s001.png]
